# Using chirality to probe the conformational dynamics and assembly of intrinsically disordered amyloid proteins

**DOI:** 10.1038/s41598-017-10525-5

**Published:** 2017-10-02

**Authors:** Jevgenij A. Raskatov, David B. Teplow

**Affiliations:** 1 Department of Chemistry and Biochemistry, Physical Science Building 356, University of California Santa Cruz, 1156 High Street, Santa Cruz, CA 95064 USA; 2 Department of Neurology, David Geffen School of Medicine, University of California Los Angeles, Los Angeles, CA 90095 USA; 3 Molecular Biology Institute (MBI) and Brain Research Institute (BRI), University of California Los Angeles, Los Angeles, CA 90095 USA

## Abstract

Intrinsically disordered protein (IDP) conformers occupy large regions of conformational space and display relatively flat energy surfaces. Amyloid-forming IDPs, unlike natively folded proteins, have folding trajectories that frequently involve movements up shallow energy gradients prior to the “downhill” folding leading to fibril formation. We suggest that structural perturbations caused by chiral inversions of amino acid side-chains may be especially valuable in elucidating these pathways of IDP folding. Chiral inversions are subtle in that they do not change side-chain size, flexibility, hydropathy, charge, or polarizability. They allow focus to be placed solely on the question of how changes in amino acid side-chain orientation, and the resultant alterations in peptide backbone structure, affect a peptide’s conformational landscape (Ramachandran space). If specific inversions affect folding and assembly, then the sites involved likely are important in mediating these processes. We suggest here a “focused chiral mutant library” approach for the unbiased study of amyloid-forming IDPs.

## Introduction

**Figure 1:**
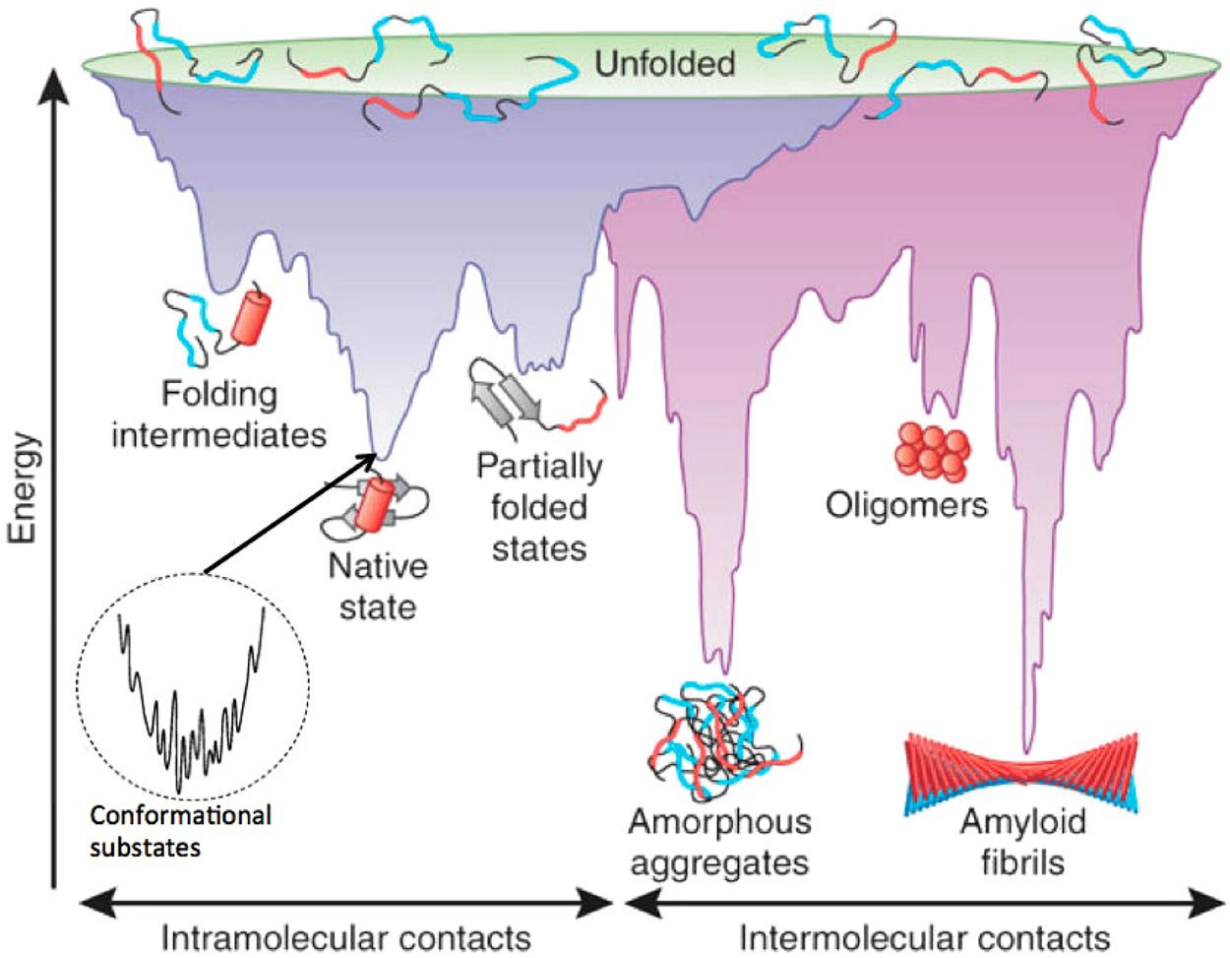
Schematic energy landscape for protein folding and aggregation. The surface shows the multitude of conformations “funneling” towards the native state via intramolecular contact formation, or towards the formation of amyloid fibrils via intermolecular contacts. The landscape is represented by the free energy of the protein as a function of some reaction coordinate (planar slices through the 3D surface). Entropy is schematized as width within any particular sub-funnel. Unfolded conformers possess the highest free energies and the largest entropies (top of funnel). Folding occurs as conformers move within (*i.e*., explore) different regions of conformational space, experiencing progressive decreases in free energy and entropy until the native state is formed. Within the minimum of the native state, a multitude of substrates exist (protein “breathing”). Figure is Fig. 1.2 of Fichou^92^, as adapted from Fig. 1 of Jahn^93^.

**Figure 2:**
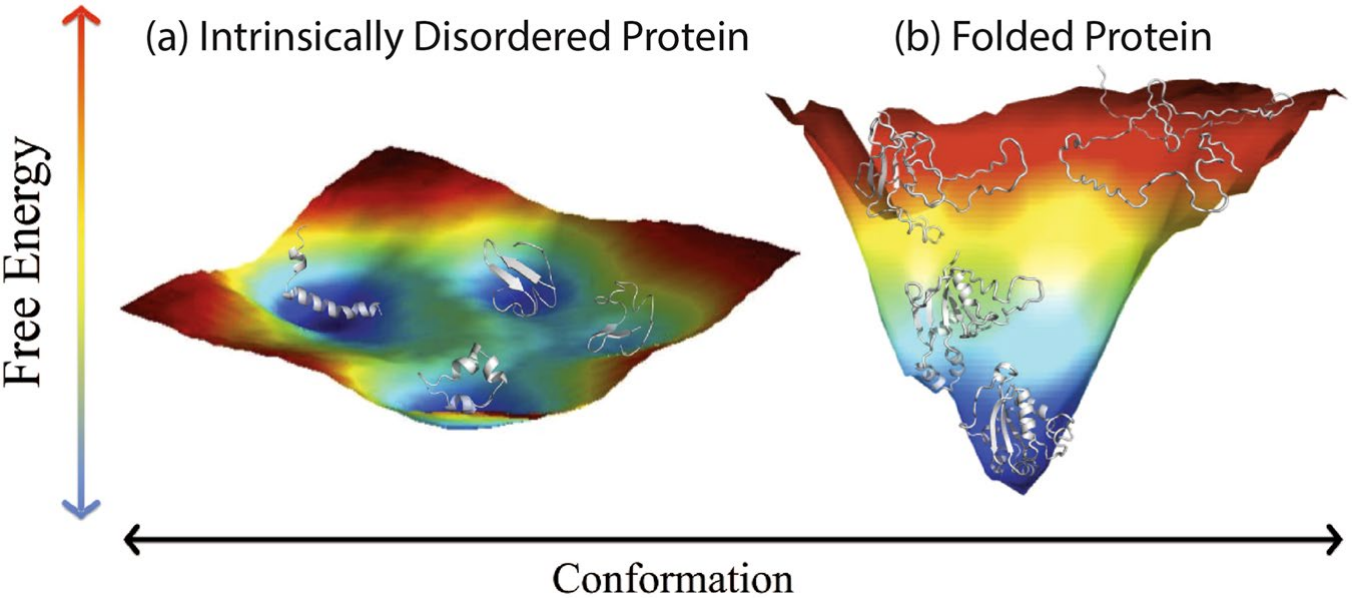
Energy landscapes. (**a**) An intrinsically disordered peptide, CcdA C-terminus. Local free energy is represented spectrally, with red highest and blue lowest. (**b**) A natively folded protein, human nucleoside diphosphate kinase (NDPK). Figure adapted from Fig. 2 of Burger *et al*.^94^.

Structure and function are inextricably linked properties of proteins. For many functions, nascent proteins must fold, after they are extruded from ribosomes, into relatively stable states of defined structure, *i.e*., their native states. However, conformational flexibility also is required for many protein functions, including enzymatic processing^1–3^, transporting cargoes^4,5^, executing regulatory functions^6^, signaling pathogen invasion^7^, and enabling neuronal communication^8^. The number of peptide backbone conformers scales approximately as 6^n^, where *n* is the number of amino acids in the peptide chain^9^. This scaling is correct if all conformers are isoenergetic. However, studies suggest that β-strand and PPII secondary structures predominate in conformational space^10–12^. This means that the actual number of conformers will be <6^n^, but nevertheless huge. This gives rise to enormous conformational diversity in unfolded proteins^13^, and if post-translational protein folding occurred *via* stochastic sampling of all possible backbone conformations, it would take longer than the age of the universe for a protein to fold into its native state (Levinthal’s paradox)^14^. The solution to this paradox is that proteins with native folds do not explore conformational space randomly. They instead undergo cooperative, sequential folding of smaller domains (foldons), thereby markedly reducing the high dimensionality of the folding process and allowing folding to occur in the millisecond time regime (Fig. 1)^15^. Foldons actually may be but one element in a larger class of small folding units that also includes inducible foldons, semi-foldons, nonfoldons, and unfoldons^16^. However, this solution is not available to the class of “intrinsically disordered proteins” (IDPs; for recent reviews, see references^17–20^) that has been suggested to display folding funnels in which: (1) wide arrays of conformers (characterized by low tertiary structure content and virtually complete absence of secondary structural elements) can populate broad, relatively flat energy regions, within which barriers to conformational sampling are extremely low (Fig. 2a); (2) initial phases of protein folding appear to involve transitions to higher energy states, in contrast to the trajectories involved in producing native folds, whose initial phases typically proceed down steep energy gradients (Fig. 2b); and (3) entropic effects contributed by solvent play an especially important role in driving folding, as opposed to enthalpic effects^21^. Such solvent-associated entropic effects also have been observed in studies of phase transitions of aqueous solutions of poly-N-isopropylacrylamide, a hydrocarbon-based polymer with peptidic side-chains that may be considered a simplified (i.e., periodic) model system for an IDP^22,23^. Below the critical temperature, hydrophobic hydration (*i.e*., solubilization of the polymer by clathrate-like encapsulation into water networks)^24^ supports the solution state. Above the critical temperature, solvent entropic gains that result from dehydration of the polymer dominate the energetics and phase separation (aggregation) occurs.

IDPs include amyloid proteins with diverse activities, such as toxicity^25^, cell compartmentalization^26^, melanocyte function^27^, and memory trace consolidation^28^. Examples of toxic IDPs are the amyloid β-protein (Aβ), α-synuclein, and amylin (islet amyloid polypeptide (IAPP)), which are seminal etiologic agents of Alzheimer’s disease (AD), Parkinson’s disease, and Type II diabetes, respectively^25^. These three peptides share the ability to generate ensembles of diverse conformational and aggregation states, the latter including oligomers, protofibrils and fibrils^29^. Establishing structure-activity relationships (SAR) among these different states, a prerequisite for proper drug targeting, is exceedingly difficult. This is a direct result of the large sizes of the conformational spaces of these proteins and the low energy barriers for traversing the space. This means that aggregation intermediates are often only present in low abundance and are associated with large numbers of assembly trajectories that result in substantial variation in monomer conformations, numbers of peptides in an aggregate, intra-aggregate organization of monomers, and shapes of the supramolecular assemblies.

Although impressive advances in our understanding of structures formed by aggregation-prone peptides have been made^30–36^, the systematic experimental characterization of structural elements as they occur in aggregation intermediates, as well as the establishment of their individual toxicity profiles, remains challenging. Due to the transient nature of these entities, as well as the concurrent formation of multiple species, structural insights can often only be obtained through *in silico* methods, calibrated against experimental observables that carry limited structural information^37–39^. Below, we introduce the theoretical framework for the use of D-amino acids as a universal tool to probe the characteristics of the folding landscapes and the associated aggregation pathways of intrinsically disordered peptides and to provide unique insights into their structure and function.

## Altering Backbone Conformational Landscapes With D-Amino Acids

We propose using D-amino acids to subtly and systematically perturb the backbone conformational landscapes of intrinsically disordered peptides. This is expected to change the relative frequencies at which the peptides follow the folding trajectories available to their wild type analogues and potentially enable exploration of new trajectories. This approach would provide new insights into the contributions of specific amino acid side-chains and segments to aggregation and toxicity. Our understanding of how the structure of the amino acid side-chain affects torsional angles that are preferentially adopted by the peptide backbone continues to improve^40–42^. Recent *in silico* studies on the model peptide systems GGXGG and AAXAA showed that the effects of amino acid chirality can be propagated through the peptide backbone without the requirement for direct interactions between the neighboring amino acid side-chains^43,44^. These (indirect) through-backbone interactions were found to be relatively subtle in terms of their perturbation to Ramachandran spaces (typically less than 10% perturbation of the original Ramachandran space in response to introduction of a D-amino acid into the model all-L-framework). Consistent with this subtle perturbation is the observation that in proteins with well-defined native structures, replacement of L-amino acids with their enantiomeric D-forms can be done without disrupting the ability of these native structures to form^45,46^. Chiral inversion even has been found to stabilize tertiary structural elements in certain cases^47–49^. In an interesting biomedically relevant case, D-substitution strategies applied to insulin have revealed that it undergoes conformational changes upon receptor binding^47^, an observation of relevance with respect to the molecular mechanisms of neonatal diabetes mellitus^50^. The chiral substitution strategy has also been previously applied to advantage in studying smaller peptides. For example, D-amino acid scanning has been used to study structural elements of the antibiotic peptide magainin 2^51^, the peptidic neurotoxin Pardaxin^52^, neuropeptide Y and corticotropin releasing factor^53^, as well as model amphipathic peptides that are prone to helix formation^54^. Identification of residues that were particularly sensitive to conformational perturbation allowed pinpointing regions with high helix-forming propensity, distinguishing them from the less structured segments of the peptide. Depending on the system (proteins, peptides) and parameter under study (e.g., structure *per se*, folding, biological activity), single D-amino acid substitutions are sufficient to cause substantial effects^55^. In some cases (e.g., HPLC retention times), di-D-amino acid substitutions are used to increase the magnitudes of the measured effects^53^. Various aggregation-prone peptides have in common that they acquire toxic properties upon association^25^. Due to the complexity of resultant aggregation manifolds^56^, the structural nature of many such toxic intermediates remains elusive^35^. The importance of such intermediates has been recognized in the field of asymmetric catalysis, where species that are readily observed due to their thermodynamic stability may not represent catalytically active species. Reaction outcomes thus can be dictated by minor (sometimes undetectable) isomers^57^. Analogous behavior may be exhibited by aggregation-prone peptides as it relates to their toxicity. For example, structure-neurotoxicity a study of Aβ40 oligomers has shown that linear increases in oligomer size produce disproportionately large increases in toxicity, suggesting that neuronal injury could be mediated by low-abundance, highly potent oligomer populations^58^. In an analogous fashion, we propose that chiral inversions could facilitate the movement of peptide conformers along folding trajectories that are rarely traversed by the wild type peptide but would lead to the formation of conformers or assemblies with especially potent neurotoxic activities. This effect might be mediated by kinetic (rates) or thermodynamic (stabilities) factors, either of which would improve the likelihood of being able to study individual steps within the trajectory through increasing the rates of formation of intermediates or stabilizing intermediates once formed.

## The “Focused Chiral Mutant Library” (FCML) Approach

How does one apply chiral inversion systematically to a peptide or protein system? The number of chiral variants that can be synthesized for a given peptide is 2^n^, where *n* is the number of chiral amino acids (all except glycine) present in the scaffold. This translates into 2^34^ = 17,179,869,184 scaffolds for Aβ40—a number far too large to be tractable synthetically in a systematic fashion. We propose a “focused chiral mutant library” (FCML) approach to obviate this problem. The FCML involves creation of a set of chiral peptide variants in which systematic chirality permutation has been applied to a small subset of the amino acids that make up the peptide of interest (selection criteria are discussed below). The focused chiral mutant library approach can be thought of as a tool to create conformationally edited scaffolds that reveal properties that are normally not readily observable (*i.e*., masked) because the corresponding conformer may not exist at sufficiently high levels, or it may exist in high levels but only transiently. The FCML approach could be applied to phenomena including the stabilization of aggregation intermediates, changes in fibrillization propensity, and modulation of biological activity.

A common feature of many disordered peptides is the gain of cytotoxic properties upon aggregation. Understanding the molecular principles that underlie the formation of such toxic entities may allow identification of small-molecule inhibitors of this process. The use of the chiral editing approach to identify conformational ensembles that have pronounced enhancement of cytotoxicity associated with them is therefore of particular interest. Selection of amino acids for the construction of a focused chiral mutant library introduces bias into the system; therefore selection criteria must be established. Selection criteria relevant to Aβ are presented below^59^.

These criteria may be extended to other aggregation-prone peptides. We note that Warner *et al*. used the hotspot strategy to identify an Aβ42 isomer with enhanced neurotoxicity^60^.

### Disease-causing mutations (hot spots)

One gene responsible for familial forms of AD (FAD) or cerebral amyloid angiopathy (CAA) is the amyloid β-protein precursor (APP) gene^61^. Mutations in APP generally produce single amino acid changes that either affect the proteolytic processing of APP into Aβ or alter Aβ primary structure directly. The latter class of mutations comprises 10 members, all but one of which (the Osaka mutation that produces Aβ missing Glu22 (ΔE22)) involve single amino acid substitutions in Aβ^62–64^. Analysis of the positions of the altered amino acids reveals two “hot spots” within the Aβ sequence that appear to be particularly relevant to disease susceptibility, Ala2-Asp6 and Ala21-Asp23. Four substitutions occur within the former region and six within the latter (four at Glu22). The fact that these single amino acid changes cause disease makes study of these sites in Aβ highly relevant and thus logical targets for FCML. A recent example of this approach was that reported by Warner *et al*.^60^, who studied the effects of a chiral inversion at Glu22 of Aβ42 on aggregation and on toxicity against PC12 cells. Mutations in APP that result in amino acid substitutions of Glu22 cause FAD (Arctic (E22G), Italian (E22K), and Osaka (ΔE22)) and cerebral amyloid angiopathy (Dutch (E22Q)). Substitution of D-Glu for L-Glu22 resulted in delayed fibrillization, with an associated 4-fold increase in toxicity of the E22e-Aβ42 variant against PC12 cells.

### Age-related epimerization

Un-catalyzed C_α_-epimerization in long-lived proteins has been well-documented for the amino acids aspartate^65^, serine^66^, and tyrosine^67^. Aspartate appears to be particularly stereolabile^68^, and epimerization of this amino acid has received significant attention^68–72^. Two FAD mutations occur at Asp residues, the Tottori mutation (D7N) and the Iowa mutation (D23N), and the influence of introducing D-aspartate at those residues has been studied because aspartate C_α_-epimerization was found to occur at those sites in Aβ extracted *post mortem* from AD brain tissues. We note that these sites could also have been chosen by selection as hot spots. *In vitro* studies have shown that epimerization at Asp1, Asp7, or Asp 23 can have substantial effects on Aβ fibrillization kinetics^73–75^. Interestingly, these alterations did not significantly alter peptide cytotoxicity, although marginal differences were noted (typically below 2-fold). It would be interesting to create a focused chiral mutant library with all possible stereoisomers, obtained through systematic variation of all stereolabile residues contained within the Aβ peptide (D1, D7, S8, Y10, D23, S26), and study the influence of these chiral edits on aggregation and neurotoxicity with both Aβ40 and Aβ42.

### “Cold spots”

The prior criteria take advantage of nature’s revelation of sites at which amino acid structural changes substantially alter protein folding, assembly, or function (hot spots). However, sites for which no mutations have been identified may also be important, and for quite a simple reason—structural changes at these sites may result in profound enhancement of, or interference with, protein function. Shirian *et al*. have referred to these sites as “cold spots,” and presented compelling experimental and computational evidence showing that amino acid changes at such sites may enhance protein function by up to six orders of magnitude^76^. These findings illustrate a very important principle, namely that restricting one’s study of structure-activity relationships *only* to sites that already have been identified through natural or experimental means may simultaneously restricts one’s ability to fully understand the dynamics and function of the protein being studied.

### Selection by theory

Advances in computational tools for simulating conformational dynamics in large systems such as proteins have enabled myriad studies of the Aβ monomer dynamics and self-association that lead to oligomerization and fibril formation (for recent reviews, see refs^77,78^). Such studies have revealed amino acids that are involved in extensive hydrogen bond networks, Coulombic and hydrophobic interactions, and backbone movements, and thus would be attractive targets for experimental structure-activity determinations. Computational approaches have the potential for bias due to the choice of starting structures, protein models (simplified, all-atom), solvent (implicit, explicit), force fields, and sampling methods. However, the ability of computational methods to pinpoint inter-atomic interactions of potential importance, interactions testable experimentally, mitigates bias and simplifies FCML execution. This criterion can be particularly powerful if applied in conjunction with one of the experimental criteria presented above, or the final criterion, presented below.

### The “no-selection” selection criterion

The most unbiased selection approach is one that has no criteria. A simple example would be the substitution of every one of the 20 naturally occurring L-amino acids at every position of Aβ, which in the Aβ40 case would produce ~20^40^ peptides, *i.e*., ~10^52^ of them. This number increases to ~10^63^ (calculated from 39^40^, because Gly is not chiral) if D-amino acids also are incorporated. A popular method for making this type of approach feasible is scanning mutagenesis. Here one *does* study every position of the peptide or protein of interest, but by substituting one chosen amino acid at only one position at a time. For Aβ40, this decreases the complexity fifty-one orders of magnitude, from 10^52^ to 40. Bias does exist because a single amino acid is substituted across the sequence. Alanine is the most common amino acid used^79–82^, but depending on the type of information desired, different amino acids have been chosen for use in scanning mutagenesis studies, including asparagine^83^, cysteine^84,85^, proline^86,87^, tyrosine^82,88,89^, and tryptophan^88,90^. Important goals of the FCML approach are to limit bias, minimize false positives, and reveal amino acids or peptide segments facilitating or inhibiting the folding and assembly events producing neurotoxic amyloid protein structures. We posit that chiral inversions allow achievement of these goals. In addition to the chiral variants described above (see FCML section, in particular), Aβ has been subjected to chiral modification of Phe19 and Phe20 to study π-π stacking^91^, but to the best of our knowledge, a systematic D-amino acid (*i.e*., chirality) scan of Aβ has not been conducted for either Aβ40 or Aβ42. An experimental study by Hayden *et al*., which accompanies this theoretical treatment of the subject, aims to close that gap^59^. In that study, a systematic analysis of the effects of chiral substitutions on Aβ conformational dynamics and assembly was done. A total of 76 peptides (32 Aβ40 and 44 Aβ42 chiral variants) were synthesized and studied. Initial screening studies were done using scanning di-D-amino substitutions. Peptides containing substitutions causing substantial effects then were re-synthesized, but with only one of the two substitutions, to determine the effects of each D-amino acid present in the initial di-peptide sequence. The analyses revealed peptide segments and specific amino acids that were particularly sensitive to D-amino acid substitution, as determined by monitoring conformational dynamics, assembly kinetics, and fibril morphology. Some sites were shared by both peptides but others distinguished Aβ40 from Aβ42.

We note parallels with experimental findings made using alanine scanning mutagenesis^80^. For example, modification of K16 or V24 of Aβ40 by alanine substitution (*i.e*., K16A and V24A)^80^ or chiral editing (*i.e*., K16k and V24v)^59^ both resulted in inhibition of fibril formation. Qualitative comparison of results obtained through our FCML approach with insights gained through other amino acid mutagenesis strategies thus may allow the pinpointing of IDP regions particularly important in facilitating or inhibiting conformational conversions to ordered structure. The value of such combined approaches is further exemplified by the fact that glycine is achiral and, hence, invisible by FCML.

## Concluding Remarks

The FCML approach provides a novel, elegant, and useful means to systematically manipulate the highly complex aggregation manifolds of IDPs. We believe that the systematic application of the FCML approach to amyloidogenic peptides, such as Aβ, α-synuclein and IAPP, has the potential to reveal valuable structure-function relationships that exist in those systems. By projecting out subsets of aggregation manifolds through stabilization of states that are only sparsely populated in the wild type (all-L) systems, insights could be gained that may enable structure-based design of therapeutic agents, targeted to disrupt specifically the toxic conformational states that are produced during IDP folding and assembly.
